# Overweight, Obesity and Influence of Stress on Body Weight Among Undergraduate Medical Students

**DOI:** 10.4103/0970-0218.55296

**Published:** 2009-07

**Authors:** Soma Gupta, Tapobrata Guha Ray, Indranil Saha

**Affiliations:** Department of Biochemistry, Midnapore Medical College, Paschim Medinipur, India; 1 Department of Community Medicine, Midnapore Medical, College, Paschim Medinipur, India; 2 Department of Community Medicine, R.G. Kar Medical College, Kolkata-700 014, India

## Introduction

Obesity is emerging as a serious problem throughout the world, not only among adults, but also children, teenagers and young adults. Of the factors contributing to obesity, stress seems to be particularly important as stressful condition leads to irregularity in diet, lack of exercise and addiction, each being considered independent factors leading to obesity.(1) Medical education is stressful throughout the whole course of training. The amount of material to be absorbed, social isolation, pressure of examination, discrepancies between expectation and reality all can be anticipated to bring psychological stress.(2) Hence, this study was undertaken to find out the prevalence of overweight and obesity among undergraduate medical students. An attempt was made to find out the significance of presence or absence of factors influencing body weight.

## Materials and Methods

A total of 114 medical students (70 male and 44 female) studying in Midnapore Medical College, Paschim Medinipur, admitted between 2004-2007, were included in this study. All the students were hostel residents of the medical college. Informed consent to participate in this study was taken from all of them and the study was approved by the ethical committee. The study period was from September 2007 to November 2007. Participants were given an information schedule and a questionnaire.

The information schedule consisted of information regarding names, age, sex, year of admission, type of diet (vegetarian/non vegetarian), dietary habit (regular/irregular), physical exercise (presents /absent) and any addiction (present/absent). The questionnaire consisted of 30 questions designed by Leveustein *et al*. (Perceived stress questionnaire). The completed form was analyzed to obtain Perceived Stress Score Index (PSSI). Higher the score index, higher will be the level of stress.(3)

Height and weight of each subject were recorded. Body Mass Index (BMI)) was calculated using the formula weight (kg)/height^2^ (m^2^) BMI less than 25 was considered normal, 25-29.9 was overweight and 30 or above obese.

Prevalence of overweight and obesity was calculated according to the standard formula (Number of cases found to be overweight or obese/number of study population ×100). Significance of non-parametric factors influencing body weight, like, type of diet, dietary habit, exercise or addiction was done by chi square test. The correlation of BMI and PSSI was done using Pearson's correlation coefficient. The proportional contribution of PSSI to influence body weight was done by multiple regression analysis. All calculations were done using statistical package, SPSS version 10.0.

## Result and Discussion

Table 1 shows the distribution of the study population and prevalence of overweight and obesity among study population. Out of 114 students, 70 were male and 44 were female (M: F = 1.6:1) Out of 70 male students, 11 were overweight and four were obese whereas out of 44 female students, nine were found to be overweight and none were obese. An overall prevalence of overweight was calculated to be 17.5%, prevalence of obesity was 3.4%. Chhabra *et al*.(4) reported a prevalence of 11.7% overweight and two per cent obesity among medical students of Delhi. Our findings are in accordance with their study. Table 2 shows distribution of subjects by factors influencing body weight. However, only the habit of exercise was found to have a significant influence on body weight of the subjects.

**Table 1:** Distribution of study population and prevalence of overweight and obesity

Sex	BMI			Total
		
	<25	25- 29.9	>30	
Male				
1^st^ year	13	4	0	17
2^nd^ year	20	2	1	23
3^rd^ year	5	2	1	8
4^th^ year	17	3	2	22
Total	55	11	4	70
Female				
1^st^ year	11	6	0	17
2^nd^ year	5	1	0	6
3^rd^ year	15	1	0	16
4^th^ year	4	1	0	5
Total	35	9	0	44

**Table 2:** Distribution of subjects based on factors influencing body weight and their significance

Body weight influencing factors	Male				Female			
		
	Normal	Over weight	Obese	Significance (*P* value)	Normal	Over weight	Obese	Significance (*P* value)
Diet type								
Vegetarian	8	2	0	NS (*P*>0.05)	6	1	0	NS (*P*>0.05)
Non vegetarian	47	9	4		29	8	0	
Diet frequency								
Regular	48	9	4	NS (*P*>0.05)	32	9	0	NS (*P*>0.05)
Irregular	7	2	0		3	0	0	
Exercise								
Present	27	3	0	S (*P*<0.05)	14	2	0	NS (*P*>0.05)
Absent	28	8	4		14	2	0	
Addiction								
Present	11	1	2	NS (*P*>0.05)	0	0	0	NS (*P*>0.05)
Absent	44	10	2		35	9	0	

Test of significance done by Chi square test, NS= Not significant, S = Significant

Researchers have reported that addition of exercise to dietary restriction can promote greater reduction in weight than change in diet alone.(5) Exercise causes nutrients to get converted to ATP based upon intensity and duration of activity initially by anaerobic metabolism. With an increase in breathing and heart rate, more oxygen is available and aerobic metabolism begins. Carbohydrates, especially glucose, are utilized first. It also causes burning of calories stored in fat. The negative caloric balance ultimately decreases body weight.(6) In our study, type of diet, frequency of diet or addiction was not found to have significant role on body weight. Studies had reported that gain in BMI was more likely to be contributed by consumption of alcohol, eating goods low in fiber, consumption of caffeine and eating cruciferous vegetables.(5)

Baker *et al*.(7) reported that the most important life style factors responsible for obesity were, long time spent using computer, eating more during time of stress and snacking between meals. In our study, significant findings were not observed which might be due to the following reasons. Due to the compact class schedule and stay in hostel, the students of this Medical College were forced to take a regular diet at least up to afternoon. They practically had little choice to select their preferred food. Moreover, a very short time is left for them to snack between meals. They do not have easy access to computer when they are in the hostel. Indian taboo and traditions prevent most of the students to smoke or consume alcohol.

The correlation between BMI and PSSI was expressed by Pearson's Correlation Coefficient. There is no gold standard for validating a measure of stress. However, PSSI have been used by other researchers as an index of stress and is now an accepted parameter for stress.(8) In case of males, positive correlative was observed which was statistically significant (r is equal to 0.362, *P*<0.01). Multiple regression analysis in males revealed that 11.8% variation of BMI was contributed by stress. In case of females, however, correlation was not found to be significant (r is equal to 0.131, *P* > 0.05). Less number of female participants might be the cause of this insignificant correlation. However, further investigation is necessary to determine the biological mechanism of stress.

The study suggests that regular exercise and taking care of stress precipitating factors could improve the health of the medical students.
